# Bridging the epistemic gap: a psychological acceptance model for the cross-cultural health communication of traditional Chinese medicine

**DOI:** 10.3389/fpubh.2026.1868985

**Published:** 2026-07-27

**Authors:** Jing Li, Ji Chen, Ping Yi, Fuyuan Lei, Yan Lv, Hong Du

**Affiliations:** School of Foreign Languages, Chengdu University of Traditional Chinese Medicine, Chengdu, China

**Keywords:** cognitive scaffolding, cross-cultural health communication, ontological insecurity, psychological acceptance, systems-oriented knowledge translation, traditional Chinese medicine, universal health coverage

## Abstract

Traditional Chinese medicine (TCM) is now included in the ICD-11, reflecting its growing use within systems of medical pluralism worldwide. Its global communication, however, continues to encounter cognitive friction. This friction reflects an epistemic gap between TCM’s holistic, pattern-based logic and the reductionist framework of biomedicine, a gap that linguistic translation cannot close on its own. The present study develops the TCM Psychological Acceptance Model (TCM-PAM) to address this challenge and to consider how the ontological insecurity often experienced by non-Chinese audiences might be alleviated. The study adopts a theory-driven, two-phase design. In the first phase, we draw on cultural schema theory, cognitive balance theory, and a systems-oriented Knowledge Translation (KT) paradigm to build a preliminary framework. The framework is then refined through consultations with a transdisciplinary panel of senior experts in TCM practice, health communication, and global health governance. The resulting TCM-PAM reconceptualizes acceptance as a three-stage cognitive trajectory: an initial stage of perception and affective filtering, a middle stage of semantic negotiation and cognitive mapping, and a final stage of internalization and identification. Three determinants are identified as central to this trajectory: perceived compatibility, cognitive cost, and trust transfer. Expert consultation suggests that pairing cognitive scaffolding such as metaphoric mapping with institutional recognition reduces ontological insecurity and supports the integration of TCM into the recipient’s evolving health identity rather than its dismissal as something foreign. By foregrounding cognitive compatibility and dynamic trust transfer, the TCM-PAM offers a framework for working across the epistemic gap through co-constructive dialog rather than unidirectional information transfer. The model speaks to a broader case for medical pluralism within global health governance, one in which integrating different medical systems rests on a serious engagement with their distinct health ontologies. Such engagement, in our view, is part of what equitable and inclusive Universal Health Coverage requires.

## 1 Introduction

Current dynamics in global health governance point to a growing engagement with traditional Chinese medicine (TCM) within systems of medical pluralism worldwide, a trend that also bears on ongoing discussions of Universal Health Coverage (UHC) (1). The World Health Organization (WHO)’s inclusion of traditional medicine in the 11th Revision of the International Classification of Diseases (ICD-11) reflects this trend (2), though it is worth being precise about what the inclusion means. The ICD-11 traditional-medicine chapter is an instrument for classification, statistics, and international comparison. It allows traditional-medicine services to be counted in a standardized way, which in turn helps generate reliable data on their safety and effectiveness. The WHO has stated that the traditional-medicine chapter “is neither judging nor endorsing the scientific validity of any Traditional Medicine practice or the efficacy of any Traditional Medicine intervention” (3). We therefore treat ICD-11 here as an indicator of the scale at which TCM is already used internationally rather than as a verdict on its clinical value; indeed, its application has been reported in more than 100 countries (4); meanwhile, the research on its pharmacological mechanisms, clinical efficacy, and international standardization (5) continues to grow. Crucially, evaluating therapeutic validity remains the domain of empirical science rather than administrative classification. In that empirical domain, the research on certain interventions has already become substantial. Acupuncture for chronic pain is the clearest case: an individual patient data meta-analysis of randomized trials, covering roughly 21,000 patients, found effects that exceeded sham treatment, persisted over time, and could not be reduced to placebo (6). Even so, interventions with this degree of support still meet difficulty once they cross cultural boundaries. The obstacle, at some point, then, lies less in the evidence than in how the underlying knowledge is understood.

This is a problem of comprehension rather than access. As discussed in the knowledge translation research (7), this friction represents the psychological pressure generated when new health information conflicts with the deeply rooted cognitive schemas of the audience. The resistance, however, is not primarily linguistic. It reflects a deep epistemic divergence: that is, the tension between TCM’s holistic and dialectical logic and the reductionist and evidence-based framework that dominates the discourse of Western biomedicine. The medical system is supported by completely different epistemic and paradigm frameworks. The interaction of different systems in cross-cultural encounters may lead to information stagnation (8) and complicate effective information exchange, especially when biomedicine conflicts with traditional explanatory models (9, 10). Against this backdrop, redundant information input is insufficient to overcome the inherent structural resistance in different health belief systems. Unless this fundamental divergence is resolved, the ongoing efforts to reconcile unfamiliar frameworks may lead to cognitive overload and a decline in engagement. Therefore, even technically precise translations may fail to achieve effective communication because they are still conceptually disconnected from the audience’s existing health belief system.

Many previous works have been conducted within the framework of the information-deficit model. It was assumed that higher terminology accuracy or wider data dissemination would linearly improve psychological acceptance (11, 12). This view, however, often overlooks the inherent cognitive friction arising from the collision of medical paradigms. In this context, the mere availability of information is not equivalent to the co-construction of meaning. Empirical observations further suggest that so-called accurate translation may itself trigger what can be described as epistemic fatigue. This term refers to a disconnection experienced by the audience when overwhelmed by conceptual barriers. Such fatigue becomes especially likely when translations impose a high cognitive load on audiences whose pre-existing cultural schemas conflict with the ontology of TCM.

We argue that this ongoing challenge originates from a gap in the existing research. Although much attention has been directed to the supply side of TCM global communication, including translated texts and export products, little is known about the demand side, particularly the psychological path followed by the audience (Table 1). Current academic research has not yet fully theorized the internal process by which non-Chinese medical consumers negotiate unfamiliar concepts such as qi or yin-yang and integrate them into their own health perception. We still lack a coherent model to explain how institutional legitimacy interacts with epistemic negotiation. This interaction serves to alleviate what sociologist Anthony Giddens calls ontological insecurity (13). In the context of TCM cross-cultural communication, this construction refers to the psychological instability that occurs when the audience’s basic health beliefs are shaken by unfamiliar medical logic and its associated cultural schemas.

**Table 1:** Research streams and the gap addressed by TCM-PAM.

Research streams	Primary focus	Identified gap	Representative references
Supply-side and standardization	International standardization, institutional policy, corpus-based representation, and the linguistic precision of TCM translation	Prioritizes technical and administrative metrics, assuming that accurate translation yields acceptance, while overlooking the recipient’s psychological responses	Wang et al. (4); Wu et al. (5); Pan (15); WHO (23)
Phenomenological barrier description	Documents the friction TCM encounters in Western societies, such as paradigm divergence and interprofessional conflict	Describes why communication is difficult, but offers no staged, mechanistic account of how audiences negotiate this friction	Anderson et al. (9); Dudla et al. (10); Li et al. (16)
Recipient-centered cognitive model	The internal cognitive and psychological processing of international audiences encountering TCM, integrating knowledge translation, cultural schemas, cognitive balance, and trust transfer	Moves past the information-deficit model to explain how recipients overcome affective, epistemic, and cognitive hurdles from initial contact to internalization	The present study [synthesizing Refs (5, 12, 15, 20) and related work]

To bridge this gap between academia and practice, this study introduces the TCM Psychological Acceptance Model (TCM-PAM). By integrating the cultural schema theory, systems-oriented knowledge translation (KT), and the principle of cognitive balance, this model does not regard the communication of TCM as the static one-way transmission of medical data. Instead, it is conceptualized as a dynamic negotiation and co-construction of meaning. Its core determines three moderating variables: cognitive cost, perceived compatibility and trust transfer. We believe that these variables are the key determinants shaping the psychological transformation a person experiences when exposed to TCM. Importantly, these mechanisms operate at two distinct decision points rather than one. They shape not only how individuals come to accept TCM therapies whose efficacy has already been established through clinical trials (application in clinical practice), but also how researchers, funders, and journal gatekeepers within Western scientific institutions determine whether TCM concepts are considered viable candidates for evidence-based investigation in the first place (application in clinical research). We return to this distinction in Section 5.2. Ultimately, this study aims to provide a theoretical basis for health communication policies that are more in line with the way humans handle unfamiliar medical concepts. In this way, true medical diversity can be supported and heal equity promoted under globalization.

## 2 Methodology: from theoretical synthesis to model refinement

**Figure 1:**
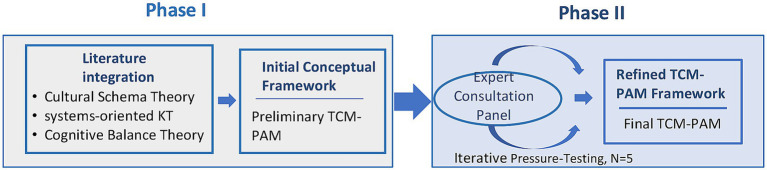
Research and validation: two iterative phases of framework development.

To ensure the theoretical soundness and ecological validity of TCM-PAM, this study adopted a two-phase research design driven by theory. As shown in Figure 1, the methodology moves from theoretical synthesis in the first phase to qualitative verification in the second phase. In Phase I, a conceptual framework is constructed by integrating three core pillars: the cultural schema theory for addressing cognitive compatibility, the systems-oriented knowledge translation (KT) for optimizing communication agency, and the cognitive balance theory for depicting the mechanism of institutional trust. This integrated framework recognizes that the global TCM communication not only requires semantic clarity but also needs to restore psychological balance through systematic legitimacy. In Phase II, iterative stress tests were conducted on the theoretical framework through semi-structured consultations with purposefully selected interdisciplinary expert groups. This approach ensures that the final model has both an ontological basis and capacity for pragmatic adaptation in response to the complexity of global health governance.

### 2.1 Conceptual framework and theoretical rationale

In this study, the cross-cultural communication of TCM was not viewed as a simple language translation exercise. Instead, it has been conceptualized as a complex cognitive negotiation. Acceptance is therefore not regarded as a binary outcome following information exposure. Rather, it is understood as a staged process of reconciling different epistemic systems. To operationalize the TCM-PAM framework, three theoretical pillars were integrated. They, respectively, correspond to the individual, communication and system dimensions.

#### 2.1.1 Cultural schema theory: assessing cognitive effort and compatibility

At the individual level, the cultural schema theory (14) indicates that the audience does not come into contact with new health information in a vacuum. They actually process this information through deeply rooted cognitive scripts. When it comes to TCM global communication, the main point of friction is schema incompatibility. Recent evidence on the international reception of TCM indicates that academic framing of its core concepts varies markedly across regions and disciplines (15), and when Western audiences are exposed to high-context concepts such as qi or meridians, they find it difficult to map these unfamiliar constructs onto existing biomedical templates (16). When the ontological distance between TCM’s holistic logic and audience’s reductionist schema becomes insurmountable, the defense mechanism often takes the lead. In this case, information may be selectively disengaged from by individuals to maintain internal consistency. By applying this perspective, we have demonstrated the rationality of taking perceptual compatibility and cognitive cost as important dimensions of the model, effectively shifting the analytical focus from information accuracy to the psychological burden imposed on the audience.

#### 2.1.2 Systems-oriented knowledge translation (KT): the agency of narrative efficacy

Traditional knowledge translation (KT) models, especially the widely cited “knowledge-to-action” (KTA) cycle (17), provide a solid structure for evidence dissemination; However, they often conceptualize knowledge as static commodities. This linear perspective is difficult to explain the complexity and social construction nature of meaning construction (18).

In contrast, the systems-oriented knowledge translation paradigm (19) holds that knowledge is a dynamic and relational process generated in a complex social system. As Best and Holmes (19) stated, their core theoretical assertion is that effective transformation requires the joint construction of meaning rather than the simple one-way dissemination of facts. This study adopts a systems-oriented KT paradigm to address the core challenge in TCM global communication: the perceptible untranslatability of its fundamental concepts. We believe that this untranslatability is usually not the failure of language, but the absence of a shared conceptual framework capable of reconciling the two paradigms (20).

To address this problem, cognitive metaphor (21) has been integrated as the core mechanism within this KT framework. Through strategic application of metaphorical mapping, for example reconceptualizing the meridian system as a dynamic information regulation network, the audience is enabled to ground the abstract philosophical principles of TCM in their familiar physical reality. Here, the systems-oriented KT paradigm shifts the focus from the objective accuracy of translating terms to the dynamic resonance of narratives. This ensures that the transformed knowledge is not only understood but also accepted as actionable and authoritative within the audience’s personal health framework.

In TCM-PAM, this process is theorized as the operational mechanism for achieving narrative efficacy. The strategic use of metaphor functions as a catalyst, actively reducing cognitive friction and enabling the joint construction of consensus understanding among different health ontologies. Therefore, narrative effectiveness is not solely an outcome. It is also a measurable process through which the epistemic gap can be bridged.

#### 2.1.3 Cognitive balance theory: negotiating ontological insecurity

Heider’s (22) classic cognitive balance theory explains how individuals seek psychological balance within interpersonal relationships. However, applying it to contemporary medical diversity requires a crucial expansion. In today’s global health landscape, trust is not only regulated through personal networks but increasingly through institutional legitimacy.

Systematic advancement of traditional medicine institutionalization has been achieved through global policy initiatives, such as WHO Traditional Medicine Strategy 2014–2023 (23). These initiatives have laid a foundation for legitimacy. On the basis of this systemic legitimacy, TCM-PAM assumes that system credibility increasingly serves as the primary anchor of public trust in complex and diversified health environments. And this institutional credibility often shapes pre-existing personal doubts (24). Such a transformation is crucial. Because the institutional validation directly alleviates the ontological insecurity that arises when individuals worry about deviation from perceived scientific safety while accessing alternative medical systems.

To formalize this mechanism, the model adapts Heider’s POX structure to what we term as POX Triad of Institutional Coordination (visualized in Section 3.1.3). It assumes that the cognitive balance of the audience (P) toward TCM (X) is achieved when the positive orientation of the trusted institution (O) toward X offsets the initial cognitive tension. Therefore, institutional support serves as a bridge for the system, reconciling macro governance authority with the micro reality of individual health practices and decision-making. It promotes trusting negotiations, enabling TCM to be integrated into an individual’s health worldview as a legal and regulated component of a diverse system, rather than being regarded as a marginal risk.

### 2.2 Expert consultation and iterative refinement

We conducted a two-round expert consultation to develop and refine the model. Through purposeful sampling, we assembled an interdisciplinary panel of five senior experts (N = 5), each with at least 15 years of experience in medical clinical practice, health communication, linguistics, and global health governance (Table 2). This composition ensured that the model’s linguistic, psychological, and systemic dimensions were each reviewed by a relevant specialist.

**Table 2:** Profiles of the transdisciplinary expert panel (*N* = 5).

Expert ID	Primary expertise	Experience	Institutional context	Role in model refinement
Expert A	TCM clinical practice and medical terminology	15 + Years	International TCM association	Reviewing clinical applicability and term accuracy
Expert B	Global health policy	20 + Years	Public health faculty	Aligning model with WHO 2014–2023 strategy
Expert C	The philosophy of linguistics	20 + Years	School of linguistics	Analyzing Cognitive Scaffolding and Epistemic Fatigue across cultures
Expert D	Health tech and AIGC	15 + Years	Digital media research lab	Assessing AIGC-driven Cognitive Scaffolding
Expert E	Critical discourse analysis and health sociology	20 + Years	Research center for health discourse and governance	Examining the mediation of institutional trust and epistemic legitimacy

In each round, experts were consulted individually through semi-structured, in-depth interviews. In the first round, they reviewed the determinants developed in Section 2.1, focusing on the proposed metaphorical-mapping and visualization tools; we then collated their feedback and revised the model accordingly. In the second round, the revised model was returned to the same experts, who examined the interaction between institutional trust and individual acceptance and assessed its consistency with current regulatory developments such as the WHO Traditional Medicine Strategy (23).

This consultation served to refine the conceptual model rather than to validate it empirically. The experts’ feedback led to two specific changes. First, AIGC-driven visual scaffolding was added as a way to alleviate epistemic fatigue. Second, institutional trust was repositioned as a core moderating variable in TCM-PAM. This aligns with growing empirical evidence on the influence of institutional trustworthiness on public attitudes and policy support (25).

## 3 Results: the TCM-PAM framework

**Figure 2:**
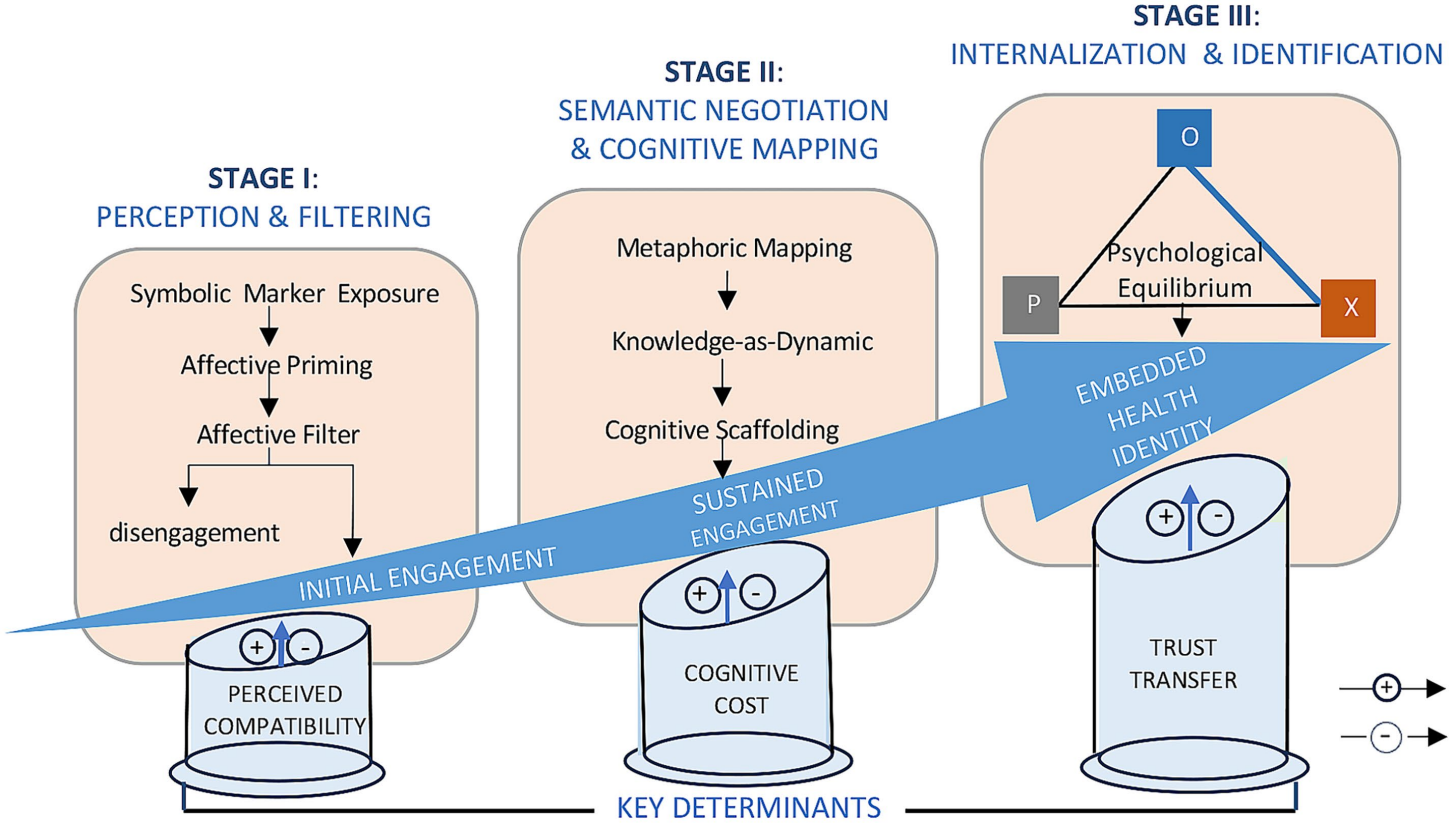
TCM psychological acceptance model (TCM-PAM).

The research findings are distilled into the TCM Psychological Acceptance Model (TCM-PAM), which is a staged framework for re-conceptualizing cross-cultural health participation. As shown in Figure 2, this model moves beyond linear information transmission and demonstrates how the audience can bridge the ontological distance between the holistic paradigm of TCM and the biomedical schema through three iterative stages (Section 3.1). Furthermore, the research identified three decisive determinants that control the success or failure of this acceptance trajectory. We believe they act as the underlying driving forces (Section 3.2).

### 3.1 The conceptual model: a three-stage trajectory of psychological acceptance

The construction of TCM-PAM adopts a process-oriented perspective, emphasizing how the audience gradually engages with, interprets and internalizes unfamiliar health knowledge. By synthesizing the insights of public health communication and cognitive linguistics, and based on the theoretical construction of deductive reasoning and expert consultation (Section 2.2), the premise of this model is that acceptance is not a static attitude outcome, but a staged cognitive trajectory. This trajectory starts from the initial information contact, undergoes active semantic processing, and eventually reaches a deep level of internalization. Although we describe these as stages for clarity of exposition, they are better understood as interacting co-factors than as a strictly sequential progression. The order presented here represents one common pathway; depending on individual disposition and the authority structure of the audience’s culture, these co-factors may overlap or occur in a different order. Where institutional authority is dominant, for instance, trust may precede rather than follow perception.

#### 3.1.1 Stage I: perception and filtering

The receiving process begins with sensory contact. Overseas audiences first encounter the symbolic signs of TCM, such as the sight of acupuncture needles, the smell of herbal prescriptions, or media images of meridian diagrams. At this point, affective priming plays a decisive role. Before any conscious assessment of clinical efficacy, these cues may trigger an immediate emotional response (26). For newcomers, this reaction is largely pre-reflective: the strangeness of the stimulus evokes emotion before thought, either inviting curiosity or prompting a defensive disconnection. But the symbols are not all received in the same way. A herbal remedy, for instance, is concrete and clinically recognizable, and so is more easily taken in and tends to invite curiosity. An acupuncture needle is similarly concrete, though for some, the needle itself may provoke apprehension rather than curiosity. Abstract ideas such as qi, meridians, or yin–yang are harder to place. They do not map neatly onto the anatomy and physiology that biomedical audiences rely on, and so more often produce unease, a sense of risk, or disengagement.

From the perspective of public health communication, this initial moment corresponds to the pre-literacy stage of health engagement, during which symbolic visibility and media frameworks crucially shape risk perception and openness to unfamiliar practices (27). Recent studies have confirmed that early emotional impressions strongly influence whether an individual continues to seek further health information or completely disengages (28), thereby initiating an implicit filtering process.

This perceptual filtering does not uniformly facilitate engagement. It is shaped by both external factors (e.g., media representation, institutional visibility) and internal factors (e.g., health status, unmet therapeutic needs) (29). When symbolic representations sharply conflict with dominant biomedical schemas, this initial filtering process may lead to what can be described as a cognitive rejection filter. At this point, further engagement is effectively terminated. In the common pathway, such initial filtering toward engagement opens the way to deeper cognitive processing, though, as noted above, this ordering is not invariant.

#### 3.1.2 Stage II: semantic negotiation and cognitive mapping

For audiences who have gone through the initial filtering, a deeper cognitive processing stage will emerge. it is characterized by the process of semantic negotiation and cognitive mapping. At this stage, individuals attempt to reconcile abstract concepts of TCM such as yin and yang, qi, and meridians, with their existing language and biomedical frameworks.

Metaphorical mapping is the main cognitive strategy, enabling the audience to transform unfamiliar philosophical constructs into more understandable entities and processes (30). This is consistent with the perspective of knowledge -as-dynamic and resonates with the co-construction of meaning, which is emphasized in the systems-oriented KT framework. It regards translation as the iterative and collaborative participation between knowledge producers and users, rather than one-way communication (31). The success of this mapping depends on health literacy. Empirical studies have shown that limited health literacy is associated with more information avoidance and selective participation, thereby amplifying cognitive burden when dealing with complex or culturally unfamiliar medical information (32, 33). Expert consultation further indicates that reconciling holistic concepts (such as five element*s*) with modern physiology is the main source of cognitive costs. When such costs become too high, epistemic fatigue will occur. Therefore, TCM-PAM believes that the second stage is not only about language translation but also about cognitive scaffolding, and targeted support is needed to maintain participation.

#### 3.1.3 Stage III: internalization and identification

The final stage marks the transition from instrumental use to value internalization. TCM is no longer perceived as an external alternative but is embedded in an individual’s health beliefs and lifestyle orientation.

**Figure 3:**
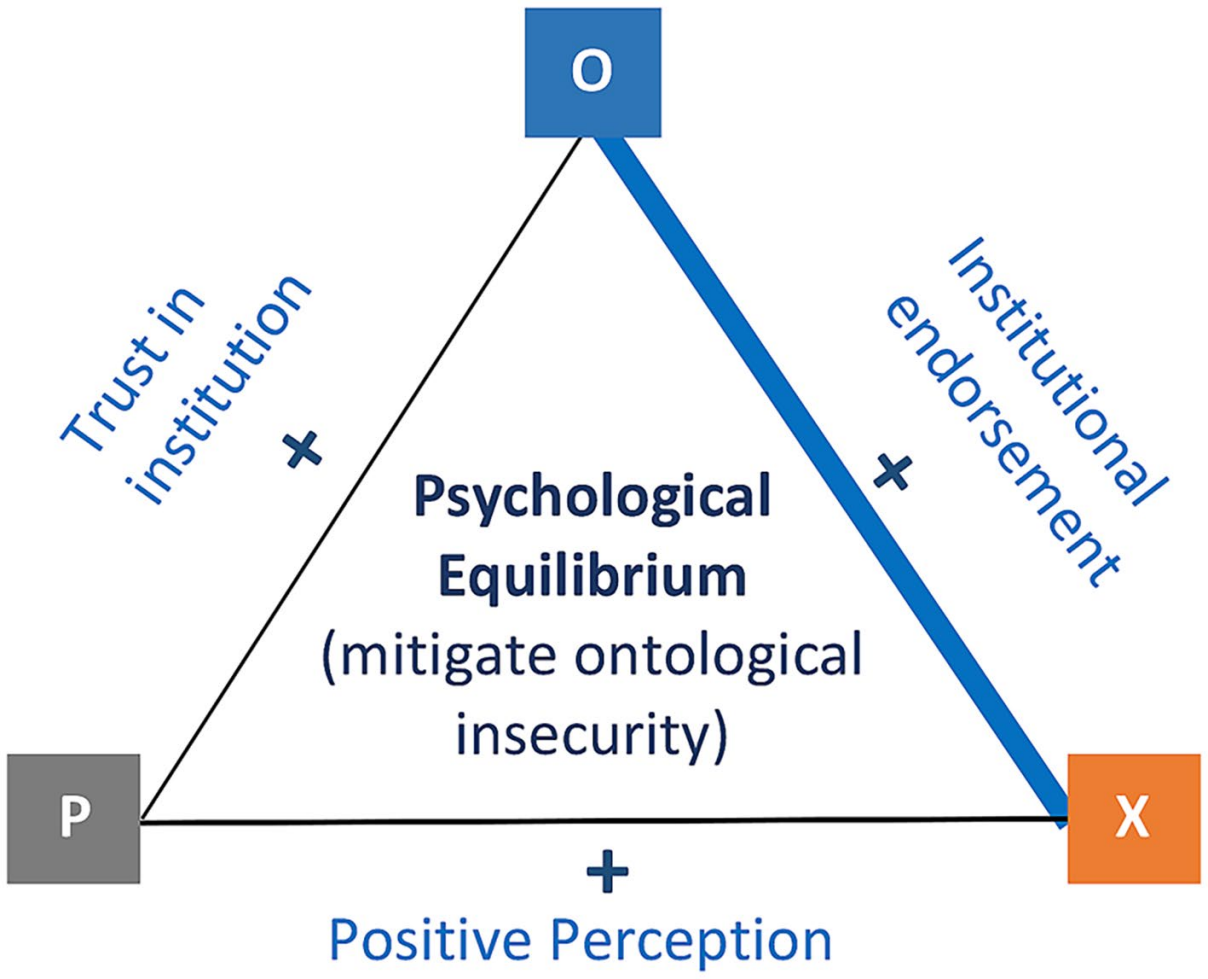
POX triad of institutional coordination.

To further decode the trust transfer mechanism discussed in Section 2.1.3, we applied the POX structure derived from Heider (22) to visualize the restoration of psychological balance. As illustrated in Figure 3, the ontological insecurities identified in early encounters are mitigated through institutional factors, which can realign the audience’s perception with the framework of less cognitive threat (34). In the POX triad, this mitigation is represented by a completely positive configuration (+ + +), that is, the trust in the institution (P-O: +), the institutional endorsement for TCM knowledge (O-X: +, highlighted), and the positive perception of TCM (P-X: +) jointly achieve a psychological balance. The research highlights that institutional support ranging from global classification systems to national regulatory frameworks and academic benchmarks has significantly reduced psychological instability associated with non-biomedical practices, as demonstrated by the inclusion of complementary and traditional medicine in ICD-11. This provides an internationally recognized classification basis and enhances the legitimacy of these systems (35). This stabilizing effect is usually a resultant force. It involves a multi-layered network of institutional factors: global and national regulations, academic benchmarks and professional certification bodies. Within the POX framework, this synergy is captured by O-X links, with institutions (O) acting as intermediary authorities to keep the audience (P) consistent in their perception of TCM knowledge (X).

This transition is supported by the experience of the decisive role of institutional support in promoting the transfer of trust. When global institutions such as the WHO and local regulatory agencies provide consistent documentation and policy recognition, they help normalize traditional medical systems and enhance their perceived legitimacy in public health infrastructure (36). Empirical evidence further proves that a higher level of institutional trust significantly lowers the psychological threshold for sustained participation and medical seeking behavior, indicating that institutional credibility plays a core role in stabilizing individual healthy participation (37). On a deeper level, institutional trust, as a social mechanism, alleviates ontological insecurity by embedding the medical system into recognized social structures, thereby enabling individuals to integrate previously unfamiliar practices into their evolving health identities (38).

### 3.2 Key determinants of psychological acceptance

Although the three-stage trajectory outlines the process of acceptance, its pace and outcome are regulated by three core determinants. These factors act as key moderating variables and play a facilitating or impeding role at the critical nodes of TCM-PAM. By integrating cultural schema theory, cognitive balance theory and the systems-oriented KT paradigm, we have identified perceptual compatibility, cognitive cost and trust transfer as the main forces controlling cross-cultural cognitive transition in health communication. Table 3 summarizes the definition of each determinant together with indicators for future empirical testing.

**Table 3:** The three core determinants of TCM-PAM: definitions and indicators.

Determinant	Definition	Indicators
Perceived compatibility	The degree to which TCM concepts and practices are perceived as resonant with the recipient’s existing cultural beliefs, life experiences, and biomedical frame of reference	Alignment with prior health beliefs; perceived consistency with biomedical knowledge; cultural/personal relevance; fit with local wellness narratives
Cognitive cost	The psychological effort and affective burden involved in interpreting unfamiliar TCM concepts through one’s existing knowledge structure	Perceived difficulty; information overload; processing fluency; uncertainty/perceived risk; epistemic fatigue
Trust transfer	The extent to which an unfamiliar medical system acquires credibility by drawing on the credibility of established institutional authorities	Trust in WHO/ICD-11 recognition; clinical recommendation; professional certification; peer testimony; perceived institutional independence

#### 3.2.1 Perceived compatibility: the filter of cultural resonance

From a public health perspective, health information must be both accurate and resonant with the audience’s life experiences and belief systems. Health communication research consistently shows that cultural beliefs and values play a core role in shaping how health information is perceived and processed. Intervention measures that fail to fit the cultural background of the target group are unlikely to be effective. This emphasizes the importance of appropriate cultural adaptation in health communication (39).

At the individual level, new evidence further indicates that participation in health information is strongly influenced by an individual’s cognitive and emotional responses. Recent work on the Health Belief Model in digital health communication suggests that engagement with health information has a lot to do with whether it aligns with the recipient’s existing health beliefs. What sustains engagement, on this account, is cognitive congruence rather than information density (40).

Cultural health communication research makes a related point. How well health information lands depend a great deal on whether it fits the audience’s cultural context, because cultural frameworks shape comprehension, emotional response, and behavior in ways that are difficult to disentangle (41). In the case of TCM, this kind of cultural fit is usually not achieved by presenting the system as an epistemic alternative to biomedicine. It tends to come instead from reframing TCM’s holistic practices, such as dietary regulation or stress modulation, in terms of wellness narratives that already resonate locally. Doing so anchors meaning-making in cultural resources the audience already draws on, which helps sustain engagement.

Proposition 1: Higher perceived compatibility between TCM philosophy and the recipient’s sociocultural and biomedical schemas increases the likelihood of activating positive cultural schemas, thereby accelerating psychological acceptance.

#### 3.2.2 Cognitive cost: the burden of epistemic translation

If compatibility is a filter, then cognitive cost is the psychological weight of negotiation. It can be understood as the psychological effort involved when an individual is required to interpret new information from the perspective of their existing knowledge structure. This becomes particularly evident in health communication when explanations are difficult to integrate with what audiences already know. Understanding such content often involves more than just simple comprehension; it also requires adjustments of the recipient’s interpretive framework, which can be a heavy psychological burden, especially when the involved concepts are unfamiliar or abstract (42, 43).

This burden, it should be noted, is not evenly distributed across audiences. Individuals with limited health literacy generally need to invest more effort to reach the same level of understanding. When this effort becomes too great, comprehension suffers and confidence in one’s own interpretation declines. Over time, such demands may produce fatigue or a gradual disengagement from health information altogether (44, 45). The problem is particularly acute in the international communication of TCM, where conceptual gaps between traditional and biomedical frameworks impose additional cognitive demands. As expert consultations in this study suggest, when audiences struggle to reconcile qi with modern physiology, they tend to respond through defensive avoidance or cognitive rejection. Once the cognitive cost exceeds what a recipient can readily process, cultural discounting becomes the easier route, even if not the more accurate one.

Proposition 2: Cognitive cost is negatively related to psychological acceptance. This cost is not purely intellectual but also affectively charged, encompassing uncertainty, perceived risk and epistemic fatigue. As the cognitive cost of integrating TCM concepts into the recipient’s existing knowledge structure rises, the likelihood of acceptance decreases.

#### 3.2.3 Trust transfer: the institutional buffer

Trust transfer refers to the process through which an unfamiliar or culturally distant medical system acquires legitimacy by drawing on the credibility of established institutional authorities. In medical pluralism, trust functions as a key determinant of risk perception and acceptance, especially when individuals are asked to evaluate health systems that go beyond their familiar biomedical framework (46).

This study conceptualizes trust as a dynamic and multi-level regulatory force that operates between institutional structures and personal life experiences. Formal support such as the WHO’s inclusion of TCM in ICD-11 provides institutional support for the exploration of alternative therapeutic options. However, this distal form of authority remains largely abstract until it is mirrored through the proximal mediation of local healthcare providers and peer networks. They help translate institutional signals into socially credible and cognitively accessible explanations in the context of daily decision-making (47). Acting as gatekeepers, they convert institutional cues into concrete, interpersonal testimonies that resonate within the local health environment. On this account, trust does not necessarily make TCM easier to understand. What it does, more precisely, is making epistemic ambiguity tolerable. By lowering the psychological threshold for engagement, trust allows recipients to temporarily hold their doubts in suspension while semantic negotiation takes place. The mechanism, it should be noted, depends on the perceived independence and credibility of institutions involved; where institutional legitimacy is itself contested, trust transfer is unlikely to operate in the same way.

Proposition 3: Trust transfer is positively related to psychological acceptance, with the combined operation of distal institutional authority and proximal interpersonal mediation lowering the psychological threshold at which recipients can sustain engagement with TCM despite epistemic dissonance.

## 4 Discussion

The conceptual framework of the TCM Psychological Acceptance Model (TCM-PAM) holds that cross-cultural TCM communication is less a linguistic transfer than a staged cognitive trajectory. The following discussion places the core propositions of the model in a broader academic conversation, clarifying its theoretical contributions at the micro, meso, and macro levels of analysis.

### 4.1 Micro-level: from information deficit to schema reconciliation

TCM-PAM poses a challenge to the common information-deficit model in health communication, which typically attributes low acceptance to a simple lack of knowledge. Our analysis indicates that more information does not automatically lead to higher acceptance. On the contrary, we propose that schema reconstruction is the key micro-mechanism at work. Although much of the existing literature on TCM translation emphasizes terminological precision, we argue that even the most accurate translations can fail if they create a prohibitive cognitive cost. When new information demands a wholesale revision of a recipient’s existing biomedical schema, the resulting mental strain often triggers defensive disengagement.

By setting perceptual compatibility as the primary filter, this study goes beyond the mechanical process of information delivery. We maintain that for a system like TCM, which has a distinct epistemology, compatibility involves functional fit as well as a state of epistemic alignment. We define it as the cognitive ability to establish a meaningful connection between new information and the existing worldview of the audience. Ultimately, this shifts the focus from how we convey information to how this information is cognitively integrated, thereby minimizing friction between medical ontologies.

### 4.2 Meso-level: from textual transmission to multimodal knowledge translation

The meso-level of TCM-PAM addresses the untranslatability of TCM by shifting from a text-centered paradigm to a multimodal mediator. We believe that traditional communication often fails because simple language translation cannot capture TCM’s profound concepts. Instead, our model regards translation as a semantic negotiation process, transforming abstract concepts into perceivable forms.

A key element of this transformation is the use of visual and sensory scaffolding. Whereas traditional approaches have relied primarily on classical texts, we emphasize the active role of technology in making abstract concepts easily perceived. AIGC (Artificial Intelligence Generated Content), for instance, functions as a cognitive scaffold that translates obscure concepts into visual narratives. By rendering complex metaphors as intuitive visual stimuli, such technologies help mitigate the cognitive strain that commonly arises in cross-cultural communication.

Through this mediation, meaning is co-constructed, and the audience is no longer a passive recipient but an active participant. Such mediation allows for embodied resonance and helps individuals understand the internal logic of TCM before fully engaging with its philosophy. Ultimately, it ensures that TCM is transformed from a static relic into a dynamic and actionable health discourse.

### 4.3 Macro-level: from cultural promotion to systemic credibility anchoring

TCM-PAM has reconceptualized institutional trust for the diversified health landscape and theorized it into a dynamic process of trust transfer. Contrary to the traditional view that trust is regarded as a static premise, our analysis demonstrates that it, as a multi-layered and mediated resource, can mitigate the ontological insecurity that individuals experience when encountering to high-friction medical systems.

A central theoretical contribution of this study lies in adapting Heider’s POX structure (22), originally a micro-level psychological tool, to the domains of macro-level governance and knowledge translation. By positioning global and national institutions as mediating authorities (O), we demonstrate how system support (such as WHO’s ICD-11) can play a role in aligning the perception of the audience (P) with that of TCM system (X). This institutional synergy establishes the legitimacy required to transform TCM from a cultural curiosity into an integral part of socially responsible health governance.

Consequently, when TCM enters discourses such as Universal Health Coverage (UHC), the relevant question shifts from whether it is an exotic alternative to how an unfamiliar and progressively EBM-tested medical tradition is understood and situated within a pluralistic health system. Ultimately, this multi-layered transfer of trust lowers the systemic psychological participation threshold, providing the necessary institutional buffer, thereby facilitating the personal acceptance journey outlined at the micro and meso levels.

## 5 Implications for cross-cultural health communication and practice

TCM-PAM offers a transformative perspective on public health communication, especially in the context of audiences with diverse cultural backgrounds and global health governance. By placing psychological acceptance at the premise of meaningful participation in medical knowledge, this study emphasizes the need to go beyond information-centered communication and shift toward cognitively-informed and affectively-grounded interventions.

### 5.1 Theoretical implications: toward an integrated science of health communication

The TCM-PAM contributes to theory by operationalizing psychological acceptance as a multi-dimensional construct. It challenges the prevailing information-deficit model, arguing that effective communication must address schema compatibility, cognitive load, and institutional trust simultaneously. This framework provides a foundation for developing an integrated science of cross-cultural health communication that is both cognitively informed and contextually grounded.

### 5.2 Practical implications for policy and communication

#### 5.2.1 Diagnosing and addressing psychological barriers

Public health initiatives should utilize the three determinants of perceptual compatibility, cognitive cost, and trust transfer as a diagnostic framework to identify points of resistance. Intervention measures must anticipate and mitigate the ontological insecurity that arises when the audience meets with unfamiliar medical paradigms, and design strategies that respect existing cognitive schemas while gently expanding the medical perspective.

#### 5.2.2 Pedagogical innovation: metaphor as a cognitive bridge

Metaphor, as we see, is not rhetoric. It belongs at the center of health education as a cognitive scaffold. Practically, this means building metaphor inventories that translate TCM concepts into more familiar frames (the meridian system as a dynamic information-regulation network, for instance). Done well, this work makes abstract philosophical content tractable, opens up entry points for public health literacy, and provides the epistemic anchoring that both community and clinical contexts require.

#### 5.2.3 Technological integration: AIGC for multimodal translation

Beyond refining technique, AIGC-driven visualization reorients Knowledge Translation toward a more systems-oriented approach. Its value is clearest with concepts that text struggles to convey. A dynamic, layered meridian animation can present the meridians as functional pathways rather than anatomical vessels, helping audiences avoid conflating them with the circulatory system; likewise, a flowing gradient can render qi as a dynamic functional state rather than a material substance. By making such concepts visible without demanding sustained reading, these tools lower the cognitive cost of entry. These tools carry risks, however. Generated visuals can oversimplify, depart from clinical or classical sources, or reinforce cultural stereotypes, and they require expert oversight to use responsibly.

#### 5.2.4 Cultivating multi-layered trust in clinical practice

Trust has to be built on two levels simultaneously. Distal legitimacy, of which the WHO’s recognition of TCM in ICD-11 is the obvious example, sets the formal stage. But proximal, expert-mediated communication in clinical encounters is what determines whether that legitimacy translates into actual engagement. Here the TCM-PAM offers a working tool: practitioners can use it to structure co-constructive dialogs, leaning on metaphor and narrative to connect TCM principles to what the patient already knows from lived experience.

#### 5.2.5 Establishing TCM as a viable object of research

A further implication sits upstream of clinical acceptance: whether TCM concepts are seen as worth studying at all. The same psychological mechanisms also shape researchers and funders. When concepts like qi or meridians clash with the methodological assumptions peer review is built on, that mismatch can make the concepts look untestable rather than simply unfamiliar; the cognitive cost of working through them can also push funding toward therapies that ask for less rethinking. Since institutional trust depends on having solid evidence, this creates a loop: TCM concepts that are not yet studied cannot generate the evidence needed to be taken seriously, and without that evidence, they have a harder time attracting the funding or publication needed to be studied in the first place. TCM-PAM therefore helps explain not just why patients accept or resist TCM therapies, but also why some TCM concepts have had a hard time entering EBM research in the first place.

In conclusion, these inspirations advocate a fundamental paradigm shift from the “one-size-fits-all,” information-centered transmission model to a cross-cultural health communication approach that is based on cognition, emotionally grounded, and supported by systems. This transformation is crucial for reducing ongoing cognitive frictions, overcoming epistemic fatigue, and ultimately achieving the goal of fair and diverse global health.

## 6 Limitations and future research

Although TCM-PAM has established a solid theoretical foundation for understanding the reception of TCM, several limitations remain, and each opens a useful direction for further research.

### 6.1 Toward empirical validation and quantifiable pathways

The most important limitation of the study is that the model has not yet been tested empirically. It is grounded in existing work on cultural cognition and knowledge translation, but it still consists of theorized pathways rather than verified ones. The proposed ordering of the model’s three stages is one such pathway: as noted in Section 3.1, it reflects one common disposition, not a fixed sequence. This is compounded by the composition of the expert panel, which was mainly domestically based and so may not fully reflect the perspectives of the non-Chinese audiences the model addresses. The next step is to operationalize the model quantitatively. Structural Equation Modeling (SEM) is well suited to this task: it would allow researchers to estimate the path coefficients linking perceived compatibility, cognitive cost, and trust transfer, and to examine how these relationships and how the ordering of the three stages, vary across cultural and demographic contexts, including among the international audiences underrepresented in the present expert panel. Without that empirical work, claims about the model’s generalizability remain provisional.

### 6.2 Situating the model within sociopolitical ecologies

Psychological acceptance of TCM is shaped by the social and political conditions in which it unfolds. National healthcare systems, media coverage, and varying levels of public exposure to complementary medicine all condition the cognitive processes described above, and their effects cannot be ignored. Future studies should therefore situate the TCM-PAM in specific institutional settings rather than treating it as a context-independent model. The relationship between health governance and individual psychological negotiation deserves particular attention. Examining this relationship more closely will help clarify how institutional permission, granted through policy recognition and regulatory endorsement, comes to operate at the level of individual health belief.

### 6.3 The efficacy and ethics of technological mediation

We have argued that AIGC-driven visualization and metaphorical mapping can reshape how TCM is communicated, but whether they actually work in clinical or community settings is still an open question. Intervention studies are needed to test how these cognitive scaffolds affect risk perception and health literacy in practice. As AIGC tools are taken up more widely in health communication, future research will also need to address the ethical questions and accuracy risks that come with AI-generated medical imagery. Visual knowledge translation that misrepresents clinical evidence or flattens the complexity of medical systems would do more harm than good. Avoiding that outcome is part of what responsible use looks like.

### 6.4 Expanding the communicative ecology

The present framework focuses on the recipient’s cognitive agency in processing and accepting TCM. It says less about the actors on the supply side, including practitioners, policymakers, and media producers, whose roles in shaping how TCM travels deserve closer attention. Future research should take a multi-stakeholder approach in order to account for the wider communicative context in which TCM knowledge moves. Bringing in the perspectives of healthcare professionals and institutional gatekeepers would reframe trust as a reciprocal relationship, and legitimacy as something co-constructed rather than conferred.

### 6.5 Generalizability and comparative medical pluralism

Finally, although this study focuses on TCM, the structure of the TCM-PAM should be useful for studying how other culturally rooted medical systems are received globally. Future research could test the model on other traditional or non-biomedical systems that encounter similar cognitive barriers when they cross cultural lines. Comparative work of this kind would sharpen the TCM-PAM and clarify how far its claims travel beyond the Chinese case. This kind of work matters for how health communication research engages with cross-cultural audiences and with medical knowledge that does not fit easily within biomedical frames.

## 7 Conclusion

### 7.1 Redefining the paradigm of acceptance

This study moves away from the view of TCM as a static cultural product and reframes its cross-cultural communication as a staged process of cognitive negotiation. The development of the TCM-PAM shows that the linguistic and cultural barriers facing TCM in global communication are neither inherent properties of the system nor fixed obstacles. They are better understood as a cognitive process that can be navigated, shaped by the interaction of cognitive cost, perceived compatibility, and trust transfer. The threshold for acceptance, in other words, is epistemic before it is anything else. What is required is a co-construction of meaning that lets TCM be understood not as something foreign imposed on the audience but as a recognizable resource for their own health.

### 7.2 Theoretical and systemic significance

The contribution of the TCM-PAM is in its synthesis. It draws on cognitive linguistics and systems-oriented knowledge translation to account for acceptance at the micro, meso, and macro levels. By taking seriously the psychological conditions under which medical knowledge is internalized, the study connects policy-level frameworks such as the WHO’s Traditional Medicine Strategy with the cognitive situations of the audiences those frameworks ultimately address. While efficacy and safety must be established through evidence-based evaluation, the framework helps explain how TCM interventions supported by such evidence may come to be understood within a pluralistic medical system rather than left at its margins.

### 7.3 Toward cognitive inclusion in universal health coverage (UHC)

The TCM-PAM also offers a way of thinking about Universal Health Coverage that takes cognitive inclusion seriously. Genuine UHC depends not only on whether diverse medical services are physically available, but on whether they are psychologically accessible and epistemically secure for the people who might use them. The model points to ways of reducing epistemic fatigue and building institutional trust across cultural lines. If such work succeeds, the integration of traditional medicine can contribute to a more inclusive form of global health governance. Different healing traditions would belong within that system, not appended to it.

## Data Availability

The raw data supporting the conclusions of this article will be made available by the authors, without undue reservation.
